# Fall risk classification with posturographic parameters in community-dwelling older adults: a machine learning and explainable artificial intelligence approach

**DOI:** 10.1186/s12984-024-01310-3

**Published:** 2024-01-29

**Authors:** Huey-Wen Liang, Rasoul Ameri, Shahab Band, Hsin-Shui Chen, Sung-Yu Ho, Bilal Zaidan, Kai-Chieh Chang, Arthur Chang

**Affiliations:** 1 Department of Physical Medicine and Rehabilitation, National Taiwan University Hospital and College of Medicine, Taipei, Taiwan, ROC; 2 Department of Information Management, National Yunlin University of Science and Technology, Douliu, Taiwan, ROC; 3 International Graduate School of Artificial Intelligence, National Yunlin University of Science and Technology, Douliu, Taiwan, ROC; 4 Future Technology Research Center, National Yunlin University of Science and Technology, Douliu, Taiwan, ROC; 5 Department of Physical Medicine and Rehabilitation, National Taiwan University Hospital Yulin Branch, Douliu, Taiwan, ROC; 6 Department of Neurology, National Taiwan University Hospital Yulin Branch, Douliu, Taiwan, ROC; 7 SP Jain School of Global Management, Sydney, Australia

**Keywords:** Falls, Machine learning, Older adults, Risk classification, Trunk sway

## Abstract

**Background:**

Computerized posturography obtained in standing conditions has been applied to classify fall risk for older adults or disease groups. Combining machine learning (ML) approaches is superior to traditional regression analysis for its ability to handle complex data regarding its characteristics of being high-dimensional, non-linear, and highly correlated. The study goal was to use ML algorithms to classify fall risks in community-dwelling older adults with the aid of an explainable artificial intelligence (XAI) approach to increase interpretability.

**Methods:**

A total of 215 participants were included for analysis. The input information included personal metrics and posturographic parameters obtained from a tracker-based posturography of four standing postures. Two classification criteria were used: with a previous history of falls and the timed-up-and-go (TUG) test. We used three meta-heuristic methods for feature selection to handle the large numbers of parameters and improve efficacy, and the SHapley Additive exPlanations (SHAP) method was used to display the weights of the selected features on the model.

**Results:**

The results showed that posturographic parameters could classify the participants with TUG scores higher or lower than 10 s but were less effective in classifying fall risk according to previous fall history. Feature selections improved the accuracy with the TUG as the classification label, and the Slime Mould Algorithm had the best performance (accuracy: 0.72 to 0.77, area under the curve: 0.80 to 0.90). In contrast, feature selection did not improve the model performance significantly with the previous fall history as a classification label. The SHAP values also helped to display the importance of different features in the model.

**Conclusion:**

Posturographic parameters in standing can be used to classify fall risks with high accuracy based on the TUG scores in community-dwelling older adults. Using feature selection improves the model’s performance. The results highlight the potential utility of ML algorithms and XAI to provide guidance for developing more robust and accurate fall classification models.

*Trial registration* Not applicable

## Introduction

Falls are one of the leading causes of accidental injuries and deaths among older individuals, and the annual incidence rate of any falls ranges between 16.5 and 32.1% among community-dwelling older individuals [1–4]. The occurrence of accidental falls is multifactorial and the combined results of multiple factors. The intrinsic factors include sociodemographic variables, physical activity, acute and chronic health problems (dizziness, cognitive impairment), mobility, alcohol consumption, and medications [3, 5, 6]. Increased numbers of risk factors are associated with an increased risk of falls, and the changes in individual conditions (such as acute illness or hazardous activities) or the presence of environmental hazard is associated with the fall risk [1]. Therefore, the prevention of falls is challenging for the complexity and dynamic nature of contributing factors.

Fall risk stratification is defined as “a single or set of assessments performed to grade an individual’s risk of falling, to guide what further assessments or interventions might be necessary” and using a standard approach to assess an individual’s estimated level of risk for falls facilitates implementation of a proportionate detailed assessment and intervention according to the level of risk [7]. Commonly used classification methods include self-reported questionnaires, physical functional tests, and posturographic parameters. Each approach has its pros and cons. For example, the Stay Independent Brochure is documented to be a valid and reliable screening tool for classifying fall risk [8], but it takes several minutes to complete the questionnaire, and there is a limitation of use in certain populations. Meanwhile, some studies recommend using physical tests, such as the Timed-Up-and-Go (TUG) test, Berg balance scale or walking speed, as screening tools [7]. However, these commonly used mobility tests require the examination of specially-trained personnel in persons and may not have sufficient discriminability to identify fallers in healthy community-dwelling older adults [9]. Another approach is to quantify an individual's intrinsic balance control using computerized posturography, which provides objective and quantitative information on body sway with no ceiling or floor effect and has the potential for autonomic recording. These posturographic parameters could be obtained under variable stance conditions to differentiate the roles of sensory input on trunk stability and provide insights into multiple aspects of postural controls [10]. These parameters are also sensitive to subtle changes of postural control, such as differentiating the control group and multiple sclerosis with minimal fall risk [11]. However, the numbers of the computed posturographic parameters may be large, and their relative sensitivity to detect changes in postural steadiness or discriminate normal versus abnormal balance controls vary considerably [11, 12]. Furthermore, these parameters may be non-linear and highly correlated [10], in which condition a traditional analysis, such as a multivariate logistic regression method, has limitations to achieving an optimal classification result. As a result, it motivates the introduction of artificial intelligence (AI)-based approach, such as machine learning (ML), to handle the complexity of the data [13].

There have been several studies combining posturographic data and AI approach for fall risk classification in several populations, including the older adults living in communities or institutes [14–18], osteoporotic elderly [19], parkinsonism [20], and multiple sclerosis [11]. The posturographic data are obtained from force platforms [11, 17, 20], pressure platforms [16], inertial sensors [16, 21], or depth camera [18]. The most commonly applied ML algorithms include random forest, decision tree, neural network, support vector machine (SVM), and k-nearest neighbor, etc. [11, 16, 17]. These various ML algorithms can achieve accuracy between 80 and 99.9% [11, 17, 22, 23], or an area under the curve (AUC) between 85 to 88% according to the receiver’s operating characteristic (ROC) analysis [16]. The above results support the validity of using posturographic features to classify or predict fall risk and may be superior to personal metrics [16]. Moreover, researchers find that feature selection for the minority class method can select previously unnoticed balance parameters, which may otherwise be disregarded by experts [19].

However, AI methods are criticized for their black-box framework nature and generally do not provide any information about the solution to the various problems and the relationships, which lead to the reliability and accountability problem [24]. It can be a significant drawback for the underlying trust issue and lead to their low use in practice, especially in healthcare [25, 26]. In response to this issue, explainable AI (XAI) techniques are proposed to describe AI behavior and present its structural and functional information clearly [27, 28]. Although the XAI results do not prove a causal relationship, it provides confidence in the model's performance by explaining how the model is derived to increase the transparency and allow the users to examine the appropriateness of the model. XAI techniques aim to provide insights into how these models make predictions or classification and to understand the reasoning behind their decisions [29]. This issue is also critical for using computerized posturographic parameters in the evaluation of fall risk. Despite the increasing application of center of pressure (COP) parameters to predict or classify fall risk in old people, the choice of COP trajectory features lacks consensus [30]. The weighting for these posturographic features to predict or classify fall risk is even more complex with inputs from the combination of personal characteristics and various postural manipulation (changing standing surface, visual input or foot positions). One study used ML algorithms and XAI approach to identify older individuals at risk of mobility decline in a 5-year follow-up [31]. Several variables are identified as important for the prediction performance according to a random forest algorithm and SHapley Additive exPlanations (SHAP) values. Therefore, introduction of XAI approach with ML algorithms have the potential to increases the transparency of models and therefore improve targeted preventive programs, which is important for fall prevention.

The goals of this study were to implement ML algorithms with posturographic parameters to classify the fall risk among community-indwelling older people. We explored the effects of feature selections on the performance and the contribution of posturographic and personal metrics on ML models according to XAI analysis. We hypothesized that the performance of the ML algorithm to classify fall risks would be better with feature selections and superior by using the TUG to using fall history as a dependent variable, since posturographic data should be correlated with the mobility ability more than the risk behaviors. The results would help to understand the relationship between changes of postural control and fall risks and to develop autonomic screening tools in the future.

## Subjects and methods

### Data collection

#### Participants and study design

This study was part of a follow-up survey of the fall risks in community-dwelling older adults. A convenience sample of elderly individuals was recruited from seven community centers, with specific inclusion criteria: being above 60 years old, living in the community, and capable of walking independently for at least 10 m, either with or without walking aids. Participants with significant cognitive impairment to follow the instructions during tests, severe visual impairment, significant neurological conditions or major musculoskeletal disorders were excluded from the study. This study was approved by the Ethical Committee of the National Taiwan University Hospital (approval number: 202112114RINA, date of approval: 1/14/2022), and written informed consent was obtained prior to participation.

#### Procedure and classification output

Two criteria were selected to categorize the fall risk. The first one was based on a single question regarding to history of fall in the previous year. The second one was according to the TUG test, which reflected mobility ability and had been commonly used to classify fall risks in the older adults [32]. All the participants were interviewed to answer a questionnaire about the personal metrics and fall history in the previous year. The information included age, sex, body weight, body height, and use of walking aids. The participants also performed the TUG test according to a standardized procedure [33], in which the participants stood up from an armed chair, walked to a line 3 m away at a safe and comfortable pace, turned, and returned to a sitting position in the chair. The time required to complete the test was recorded in seconds by a stopwatch.

#### Data collection with a VIVE tracker-based posturography

**Fig. 1:**
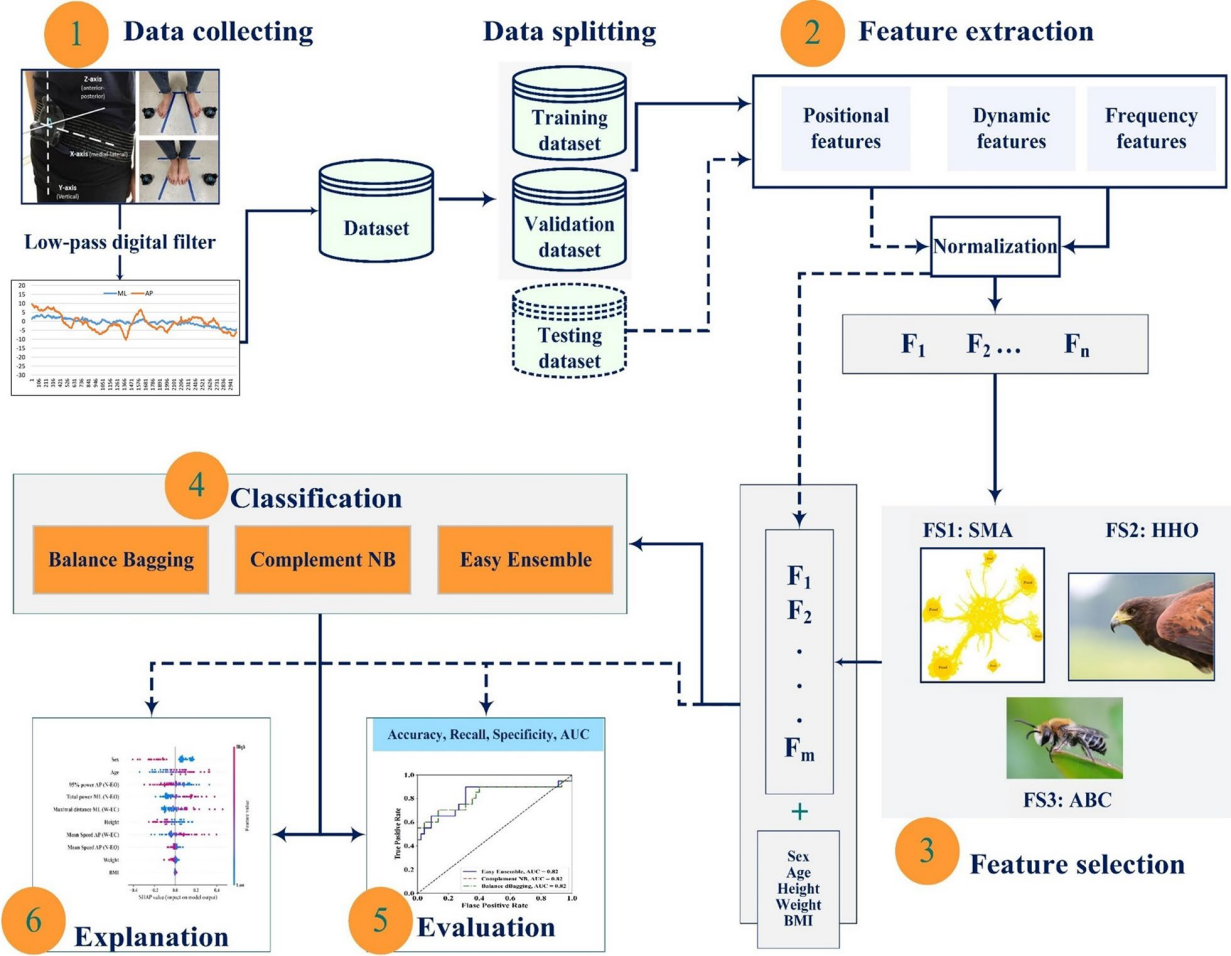
Proposed method for machine learning

We obtained body sway parameters through a VIVE (HTC, Inc. Taiwan) tracker-based posturography. The setup included two infrared laser emitter units (SteamVR Base Stations V2.0) and three wireless trackers (Steam VR Tracking V2.0). The details of the setup and the reliability and validity against a platform system had been given in previous study [34]. In brief, trunk displacement trajectories were obtained from one tracker positioned on the posterior lumbar region at the pelvic level with a reference body frame established by two trackers put on the floor and lateral to the feet (Fig. 1). The time series of trunk displacements of the lumbar tracker (TD_L_) were recorded in the medial–lateral (M-L) and anterior–posterior (A-P) directions from the VIVE tracker-based posturography as a proxy of trunk sway near the level of the center of mass [35], and to compute TD_L_ parameter as input for ML.

The participants were informed of the procedure first and then performed bipedal stance under four conditions: feet apart with eyes open (W-EO), feet apart with eyes closed (W-EC), feet together with eyes open (N-EO), and feet together with eyes closed (N-EC). During the standing tasks, the participants were instructed to put their arms down at their sides and remain as stable as possible for 30 s. All the standing tasks were standardized with marking on the floor (Fig. 1). In the EO stance, the participants were asked to look at a fixed target on the wall 2 m ahead. The testing order of the four conditions was randomized, and each posture had two trials, yielding a total of eight bi-axis trajectory data for each participant.

The time series of the TD_L_ data were passed through a fourth-order zero-phase Butterworth low-pass digital filter with a 5-Hz cutoff frequency and used to compute 31 parameters (Table 1) as the input of ML [10]. These parameters were grouped as positional, dynamic, and frequency measures, and the computation was based on the equations used for COP trajectory analysis in previous studies [10, 12].

**Table 1:** A complete list of the posturographic parameters for feature extraction

Parameters	Directions
Time-domain distance measures	
Mean distance	M-L, A-P, radius
Maximal distance	M-L, A-P, radius
Root mean square distance	M-L, A-P, radius
Range	M-L, A-P, radius
Mean velocity	M-L, A-P, radius
Time-domain area measures	
95% confidence ellipse area	radius
Time-domain hybrid measure	
Sway area per second	radius
Mean frequency	M-L, A-P, radius
Fractal dimension	radius
Frequency domain measure	
Total power spectrum density	M-L, A-P
50% power frequency	M-L, A-P
95% power frequency	M-L, A-P
Centroid frequency	M-L, A-P
Frequency dispersion	M-L, A-P

We used the computation for center of pressure trajectory analysis [10, 12]

M-L: medial–lateral; A-P: anterior–posterior

## Methodology of machine learning

Figure 1 presents an overall framework to classify fall risks, which includes data collection, filtering, feature extraction, selection, and classification. The evaluation process is based on several metrics, and the result is explained using the SHAP method [36]. More information regarding each step is provided as follows:

### Feature extraction

In this step, we computed 31 features from the TD_L_ trajectories according to previous studies (Table 1) [10, 12]. Personal metrics (age, sex, height, weight, and body mass index (BMI)) were also considered as features since they are either risk factors for falls or might influence the data recorded by a lumbar tracker. Each participant performed two trials for each stance condition (W-EO, W-EC, N-EO, N-EC), and the parameters from the two trials were averaged for each stance condition. There were a total number of 124 features from posturographic data. The filtered data were split into three sets: training (50%), validation (20%), and test sets (30%). Also, three-fold cross validation methods on training data were used for parameter setting. In the next step, we applied feature selection methods to improve the training time and accuracy of the models. Finally, the extracted features were normalized between 0 and 1 on the training set and test set.

### Feature selection

Feature selection is a widely used technique in ML that serves to speed up the training time, improve the accuracy of models, and reduce overfitting [37]. These feature selection methods also helped us identify the most significant and relevant posturographic parameters as features for fall risk classification, thereby improving the accuracy of the classifiers, and resulting in a robust solution for fall risk classification.

We selected three meta-heuristic algorithms, Harris Hawk Optimization (HHO) [38, 39], Slime Mould Algorithm (SMA) [40], and Artificial Bee Colony (ABC) [41] to enhance the quality of features for classification. HHO is a meta-heuristic optimization algorithm that is inspired by the hunting behavior of Harris hawks. The algorithm is designed to solve optimization problems with continuous and discrete variables. One of the key advantages of HHO is its simplicity and robustness. Additionally, it has the ability to efficiently search in high-dimensional feature spaces. Also, it is efficient in avoiding getting trapped in local optima and can adapt to different types of optimization problems [38, 39]. Another feature selection method is based on SMA which is a meta-heuristic algorithm based on the oscillation mode of slime mold in nature. This algorithm is designed for engineering problems and continues global optimization and has shown reasonable performance for feature selection in previous research [42, 43]. The third feature selection method employed is ABC, which is based on ants' behavior in finding food. This algorithm is used as an optimization technique, demonstrating that a reduced number of features can achieve classification accuracy superior to that obtained using the full set of features [41].

#### Fitness function

The results of the classification were then evaluated using metrics such as accuracy, sensitivity, specificity, geometric mean (GM), and area under the curve (AUC). In feature selection, the fitness function is typically defined as the accuracy or performance of a classifier trained on the selected features. However, the combination of AUC, GM, and the size of selected features is considered a fitness function according to previous research [44]. The proposed fitness function is defined as:

1 $$\mathrm{fitness }={{\text{w}}}_{1}\mathrm{GM }+ {{\text{w}}}_{2}\mathrm{AUC }+ {{\text{w}}}_{3}\uplambda$$

where $\uplambda =1-(\frac{\left|{X}^{*}\right|}{\left|X\right|})$ where $\left|{X}^{*}\right|$ represents the size of selected features and $\left|X\right|$ denotes the size of input features, and AUC denotes the area under the curve, and GM is calculated as:

$${\text{Sensitivity}}=\frac{TP}{TP+FN}$$

$${\text{Specificity}}=\frac{TN}{TN+FP}$$

2 $${\text{GM}}=\sqrt{{\text{Sensitivity}}\times {\text{Specificity}}}$$

where TP denotes “true positives” which is the number of samples that are correctly classified as positive by the model, FP represents “false positives” which are the number of samples that are incorrectly classified as positive by the model. TN stands for “true negatives” which are the number of samples that are correctly classified as negative by the model. FN represents “false negatives” which are the number of samples that are incorrectly classified as negative by the model Hossin and Sulaiman [45].

### Classification

The selected features based on the three feature selection methods of SMA, HHO, and ABC, along with several personal metrics, were then used for classification using three different algorithms: Easy Ensemble [46], Balanced Bagging [47] and Complement Naïve Bayes (NB) [48]. Easy Ensemble creates an ensemble of classifiers by under-sampling the majority class, Balanced Bagging by re-sampling the training data with replacement, and Complement NB by combining the predictions of multiple Naive Bayes classifiers trained on different subsets of the feature space. Since the annual prevalence of any falls was mostly under 35% among community older adults [1, 3], an imbalanced data set was expected. The above algorithms were suitable for imbalanced data classification to improve classification performance by reducing bias toward the majority class [46]. We also compared these methods with traditional approaches for feature selection and classifiers. Two criteria were used for the classification: criteria I: any fall in the previous 1 year, and criteria II: requiring 10 s or more to complete the TUG test. Criteria II reflected the current ability of balance. The cutoff value of the TUG test was lower than that proposed by a recent guideline [7], but was considered of good predictive validity of fall risks in community-dwelling older adults [4]. Previous studies also showed an inadequate discriminative ability using 13.5 s [49].

### Evaluation metrics

In this study, various evaluation metrics were used to assess the performance of our proposed model. These included accuracy, sensitivity (recall), specificity, and the AUC [45]. These metrics provide a comprehensive evaluation of the model's performance, providing valuable insights into its capabilities. The overall performance of the model is deemed to be satisfactory when it exhibits high levels of accuracy, sensitivity, specificity, and AUC.

### Explainability

Lastly, the results of the evaluation were explained using the SHAP method [36]. This method provides an understanding of how different features contribute to the final classification result [31, 50], providing insight into the importance of each feature to classify fall risks accurately in older adults. SHAP is based on Shapley values, which explain individual weights from a model. Shapley values are defined as a coalition game with players and a value function. In the ML approach, the 'players' represent input variables or features, and the 'game' represents the prediction of the machine learning models. Shapley values were introduced to fairly distribute the payout of a cooperative game among its players. Shapley values assign a value to each feature by considering its contribution to different possible coalitions of features.

## Results

### Collected data of the participants

We recruited 217 participants, but two of them were not able to complete the fourth task, N-EC. Therefore, only data from 215 participants were included for analysis. They were, on average, 72 years old, and around one-third of them were female. The fall rate in the previous year was 22.8%, with 32.7% of the fallers falling more than once. The proportion of the participants classified as at risk was higher with Criteria II (30.7%) than with Criteria I (22.8%). With criteria I, the at-risk group had a higher proportion of using walking aids, but similar to low-risk group with other personal metrics and TUG scores. With criterion II, the comparison between the low-risk and at-risk groups showed a significant difference regarding age, body height, BMI, using walking aids and having multiple falls (Table 2).

**Table 2:** Personal metrics of all participants and comparison between the low-risk and at-risk groups according to two criteria: criteria I: according to any fall in the past 1 year, criteria II according to the TUG test

	Criteria I			Criteria II		
	Low risk (N = 166)	At risk (N = 49)	p value	Low risk (N = 149)	At risk (N = 66)	p value
Age	71.2 ± 7.3	72.6 ± 6.7	0.24	70.0 ± 6.6	75.3 ± 7.0	< 0.001
Male	53 (31.9%)	16 (32.7%)	0.92	49 (32.9%)	20 (33.3%)	0.71
Body height (cm)	156.9 ± 8.7	157.4 ± 8.5	0.76	158.2 ± 7.8	154.3 ± 9.7	0.002
Body weight (kg)	61.0 ± 12.5	62.3 ± 19.6	0.50	61.1 ± 12.1	61.7 ± 12.1	0.73
Body mass index (kg/m^2^)	24.7 ± 4.1	25.2 ± 4.2	0.40	24.3 ± 4.0	25.9 ± 4.1	0.01
Using walking aids	11 (6.6%)	8 (42.1%)	0.04	0 (0%)	19 (28.8%)	< 0.001
Timed-up-and-go time (seconds)	8.5 ± 2.9	9.0 ± 3.6	0.28	7.5 ± 1.1	12.8 ± 4.0	< 0.001
Fall history						
Any fall	–	–	–	21 (14.1%)	28 (42.4%)	< 0.001
Multiple falls	–	–	–	3 (2.0%)	13 (19.7%)	< 0.001

### Initialization and parameters setting

Table 3 shows the hyperparameters for three classifiers used for classification, which include Easy Ensemble, Balanced Bagging, and Complement NB. For the Easy Ensemble classifier, the number of AdaBoost learners in the ensemble is set to 9, and the estimator used to grow the ensemble is Complement NB. The Balanced Bagging classifier has a hyperparameter for the number of base estimators in the ensemble, which is set to 9, and uses Complement NB as the base estimator to fit on random subsets of the dataset. The hyperparameter for this classifier also includes a setting for whether features are drawn with replacement, which is set to True. Finally, the Complement NB classifier has an additive smoothing parameter, which is set to 1.0. These hyperparameters determine the behavior of the models and can impact their performance on the classification. Also, the population size and epoch for three feature selection models based on meta-heuristics, SMA and HHO, have been set to 100 and 100, respectively. All simulations were carried out in Python 3.8 using the scikit-learn and mealpy packages on a machine equipped with a Core i7 processor running at 2.70 GHz and 32 GB of memory.

**Table 3:** initialization parameters

Classifiers	Hyperparameter	Values
Easy Ensemble	Number of AdaBoost learners in the ensemble	9
	Estimator used to grow the ensemble	Complement NB
Balanced Bagging	The number of base estimators in the ensemble	9
	The base estimator to fit on random subsets of the dataset	Complement NB
	Whether features are drawn with replacement	True
Complement NB	Additive (Laplace/Lidstone) smoothing parameter	1.0
SMA, HHO, ABC	Population size Epoch	100 100

HHO: Harris Hawk Optimization; SMA: Slime Mould Algorithm, ABC: Artificial Bee Colony

### Classification results

**Fig. 2:**
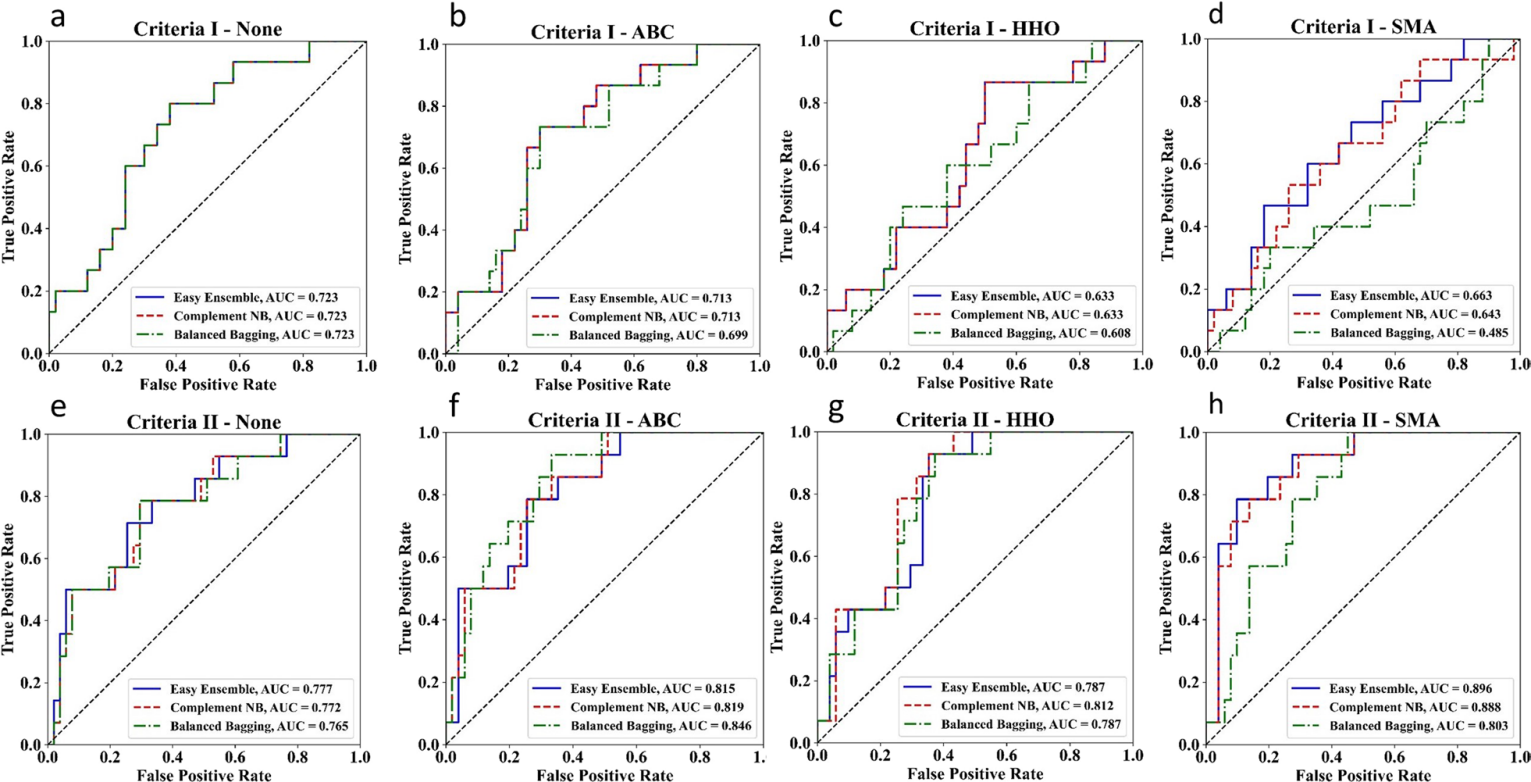
The plots of receiver operating curve analysis according to different classification criteria, feature extraction and classifiers. (ABC: Artificial Bee Colony; HHO: Harris Hawk Optimization; SMA: Slime Mould Algorithm)

Tables 4 and 5 present the results of different combinations of feature selection models (SMA, HHO and ABC) and classifiers (Balanced Bagging, Complement NB, and Easy Ensemble) for a fall risk classification using two criteria. The evaluation metrics include accuracy, recall (sensitivity), specificity, and AUC. Overall, using Criteria II achieved higher accuracy (0.66 to 0.78) and AUC (0.76 to 0.90) than using Criteria I. The highest AUC was achieved by using Criteria II and the SMA feature selection model with easy Ensemble. The Complement NB classifiers yielded better performance in terms of accuracy, recall, and specificity compared to the Balance Bagging with Criteria II. However, feature selection resulted in higher accuracy compared to not using feature selection with only Criteria II, but not Criteria I. Figure 2 displays the AUC plot for three feature selection approaches, followed by classification using the Balanced Bagging, Complement NB, and Easy Ensemble classifiers.

**Table 4:** Output using Criteria I, at risk of fall according to fall history in the past 1 year, as a classification criterion

Feature selection	Model	Accuracy	Recall	Specificity	AUC
ABC	Balanced Bagging	0.69	0.73	0.68	0.70
	Complement NB	0.69	0.67	0.70	0.71
	Easy Ensemble	0.69	0.67	0.70	0.71
HHO	Balanced Bagging	0.60	0.47	0.64	0.61
	Complement NB	0.60	0.40	0.66	0.63
	Easy Ensemble	0.60	0.40	0.66	0.63
SMA	Balanced Bagging	0.58	0.40	0.64	0.49
	Complement NB	0.62	0.53	0.64	0.64
	Easy Ensemble	0.60	0.60	0.60	0.66
None	Balanced Bagging	0.71	0.60	0.74	0.72
	Complement NB	0.71	0.60	0.74	0.72
	Easy Ensemble	0.71	0.60	0.74	0.72
Mean	Balanced Bagging	0.65	0.55	0.68	0.63
	Complement NB	0.66	0.55	0.69	0.68
	Easy Ensemble	0.65	0.57	0.68	0.68

AUC: area under the curve; ABC: Artificial Bee Colony; HHO: Harris Hawk Optimization; SMA: Slime Mould Algorithm

**Table 5:** Output using Criteria II, scores 10 s or more with the TUG test, as a classification criterion

Feature selection	Model	Accuracy	Recall	Specificity	AUC
None	Balanced Bagging	0.69	0.57	0.73	0.76
	Complement NB	0.69	0.57	0.73	0.77
	Easy Ensemble	0.69	0.71	0.69	0.78
ABC	Balanced Bagging	0.74	0.86	0.71	0.85
	Complement NB	0.75	0.71	0.76	0.82
	Easy Ensemble	0.72	0.79	0.71	0.82
HHO	Balanced Bagging	0.71	0.64	0.73	0.79
	Complement NB	0.72	0.64	0.75	0.81
	Easy Ensemble	0.66	0.57	0.69	0.79
SMA	Balanced Bagging	0.72	0.64	0.75	0.80
	Complement NB	0.78	0.86	0.76	0.89
	Easy Ensemble	0.77	0.86	0.75	0.90
Mean	Balanced Bagging	0.72	0.68	0.73	0.80
	Complement NB	0.74	0.70	0.75	0.82
	Easy Ensemble	0.71	0.73	0.71	0.82

AUC: area under the curve; ABC: Artificial Bee Colony; HHO: Harris Hawk Optimization; SMAl: Slime Mould Algorithm

**Fig. 3:**
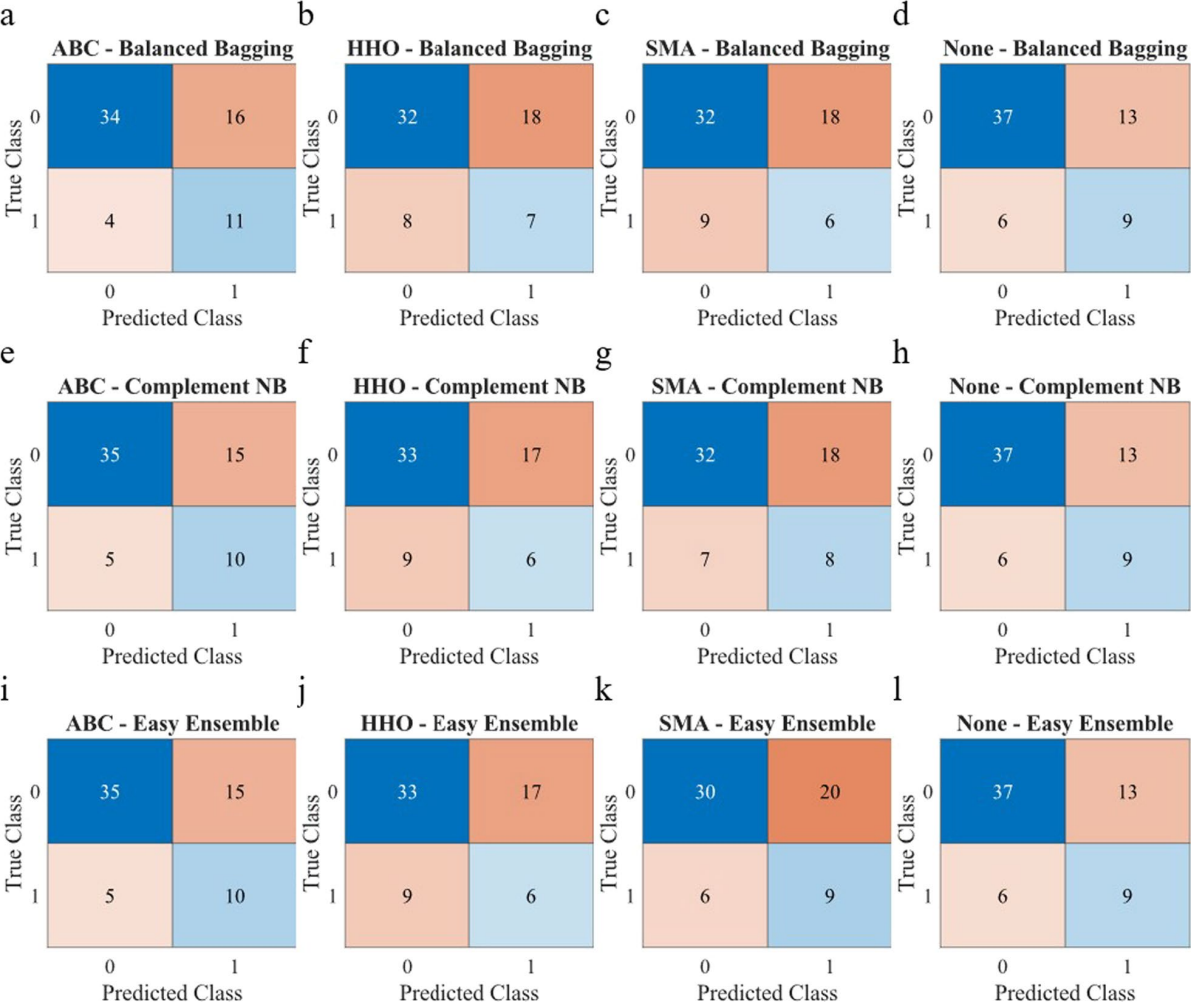
Confusion matrices for different feature selections and classifiers for Criteria I. It displays the number of true negatives (TN) at the left upper corner, true positives (TP) at the right lower corner, false positives (FP) at the right upper corner, and false negatives (FN) at the right lower corner, according to the model's predictions (ABC: Artificial Bee Colony; HHO: Harris Hawk Optimization; SMA: Slime Mould Algorithm)

**Fig. 4:**
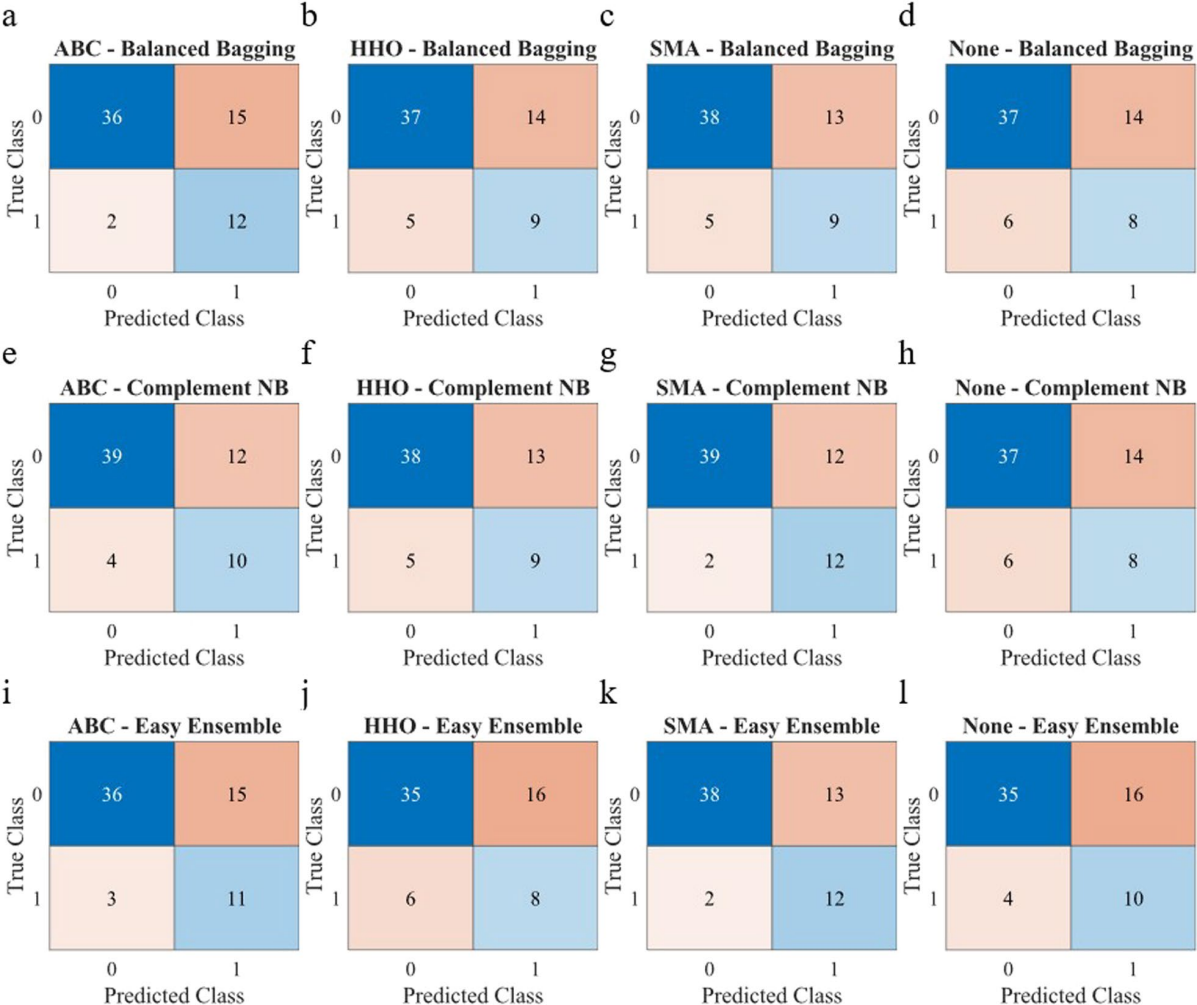
Confusion matrices for different feature selections and classifiers for Criteria II. It displays the number of true negatives (TN) at the left upper corner, true positives (TP) at the right lower corner, false positives (FP) at the right upper corner, and false negatives (FN) at the right lower corner, according to the model's predictions. (ABC: Artificial Bee Colony; HHO: Harris Hawk Optimization; SMA: Slime Mould Algorithm)

Figures 3 and 4 demonstrate the confusion matrices for fall risk classification based on Criteria I and II, respectively. In Criteria I (Fig. 3), Easy Ensemble classifiers using the selected features by ABC stand out with the highest TP, correctly identifying individuals at risk of falling, while Balanced Bagging without any feature selection algorithm achieves the highest TN, accurately classifying those not at risk. On the other hand, Balanced Bagging with ABC and Easy Ensemble with SMA achieve the lowest FP and FN, indicating superior performance in minimizing misclassifications. In Criteria II (Fig. 4), Balanced Bagging with ABC algorithm obtains the highest TP, effectively identifying individuals at risk, while Balanced Bagging classifier without any feature selection algorithm maintains the lead in TN, providing accurate predictions for those not at risk. The lowest FP is achieved by Balanced Bagging and Easy Ensemble with ABC, emphasizing their effectiveness in avoiding false alarms, and Complement NB with SMA achieves the lowest FN, showcasing its strength in minimizing missed fall risk classifications.

### Comparison with traditional feature selection methods

**Fig. 5:**
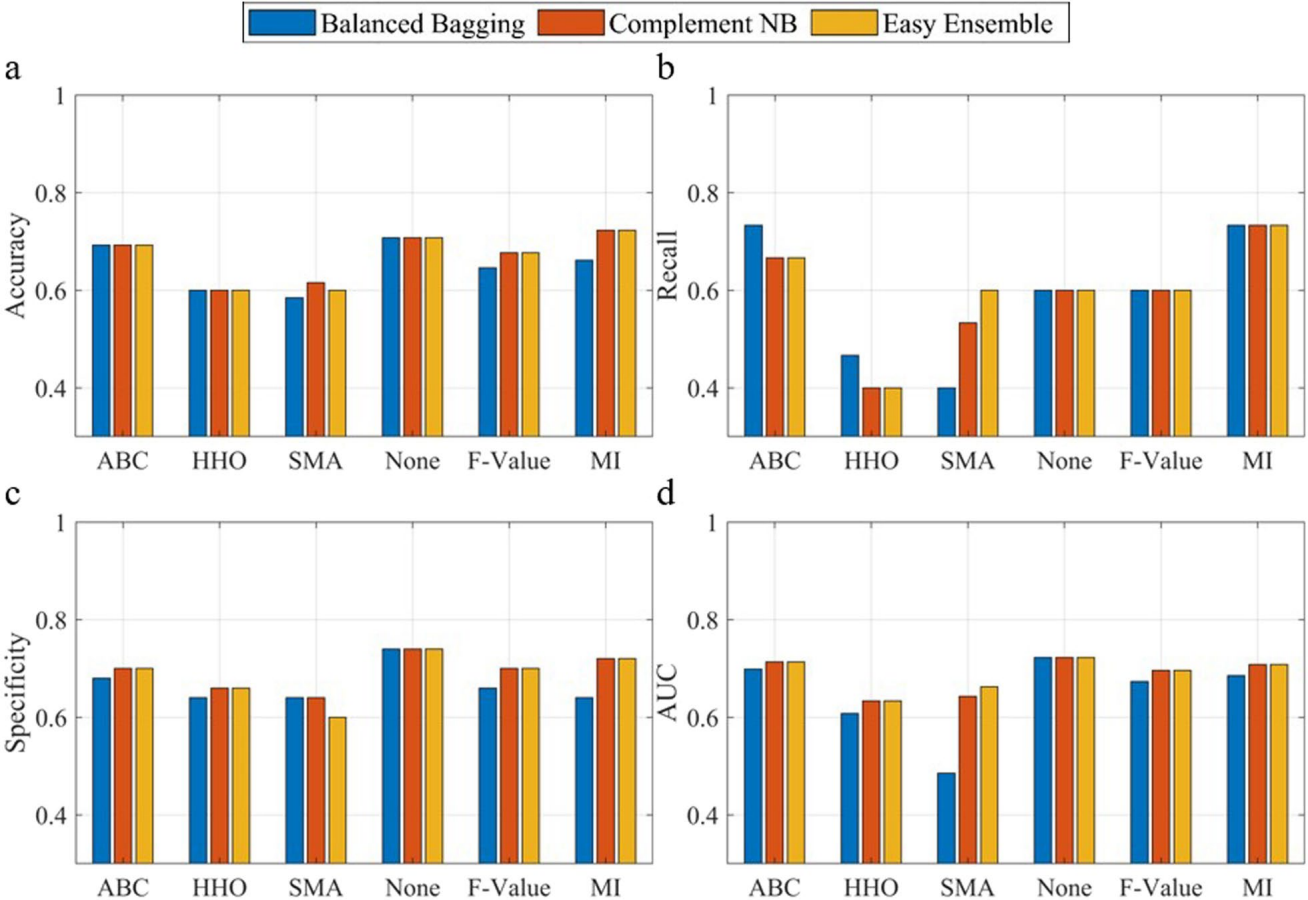
Model performance comparison using various feature selection methods for criteria I, illustrated for accuracy (**a**), recall (**b**), specificity (**c**) and area under the curve (**d**). (ABC: Artificial Bee Colony; HHO: Harris Hawk Optimization; SMA: Slime Mould Algorithm; MI: Mutual Information)

**Fig. 6:**
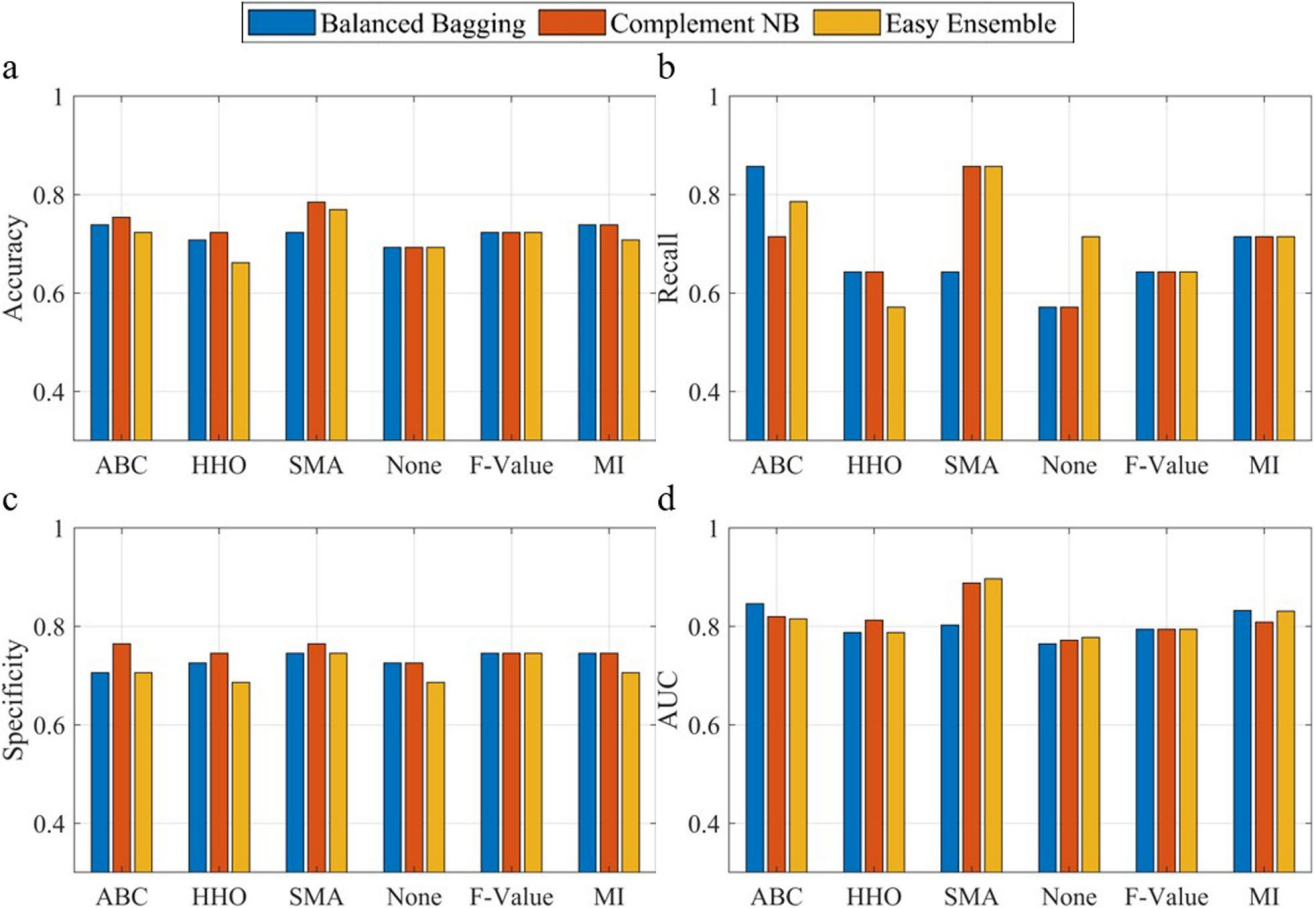
Model performance comparison using various feature selection methods for criteria II, illustrated for accuracy (**a**), recall (**b**), specificity (**c**) and area under the curve (**d**). (ABC: Artificial Bee Colony; HHO: Harris Hawk Optimization; SMA: Slime Mould Algorithm; MI: Mutual Information)

Figures 5 and 6 demonstrate the comparative analysis of fall risk classification results obtained through traditional feature selection methods, specifically Mutual Information (MI) [51] and ANOVA F-value (F-value), combined with three classifiers (Balanced Bagging, Complement NB, and Easy Ensemble). In Criteria I (Fig. 5), results obtained for ABC, HHO, and SMA consistently outperform traditional feature selection methods such as MI and F-value across various metrics, including accuracy, recall, specificity, and AUC. This trend holds true for Criteria II (Fig. 6), where metaheuristic algorithms ABC, HHO, and SMA showcase superior performance compared to traditional methods, emphasizing the effectiveness of the selected algorithms in enhancing fall risk classification accuracy.

### Comparison with traditional classifiers

**Fig. 7:**
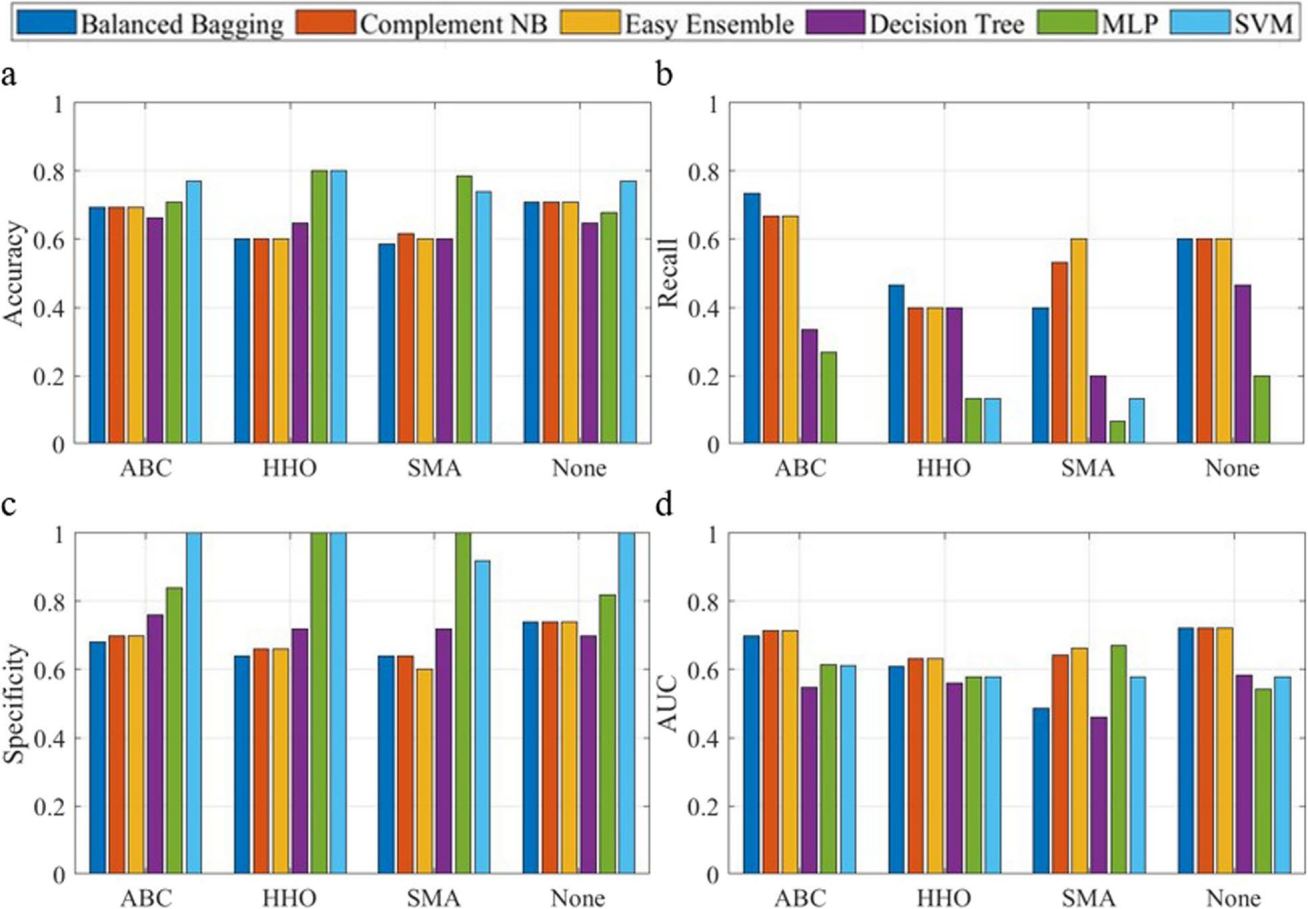
Model performance comparison using various classifiers for criteria I, illustrated for accuracy (**a**), recall (**b**), specificity (**c**) and area under the curve (**d**). (MLPl: Multi-layer Perceptron; SVM: Support Vector Machine; AUC: area under the curve)

**Fig. 8:**
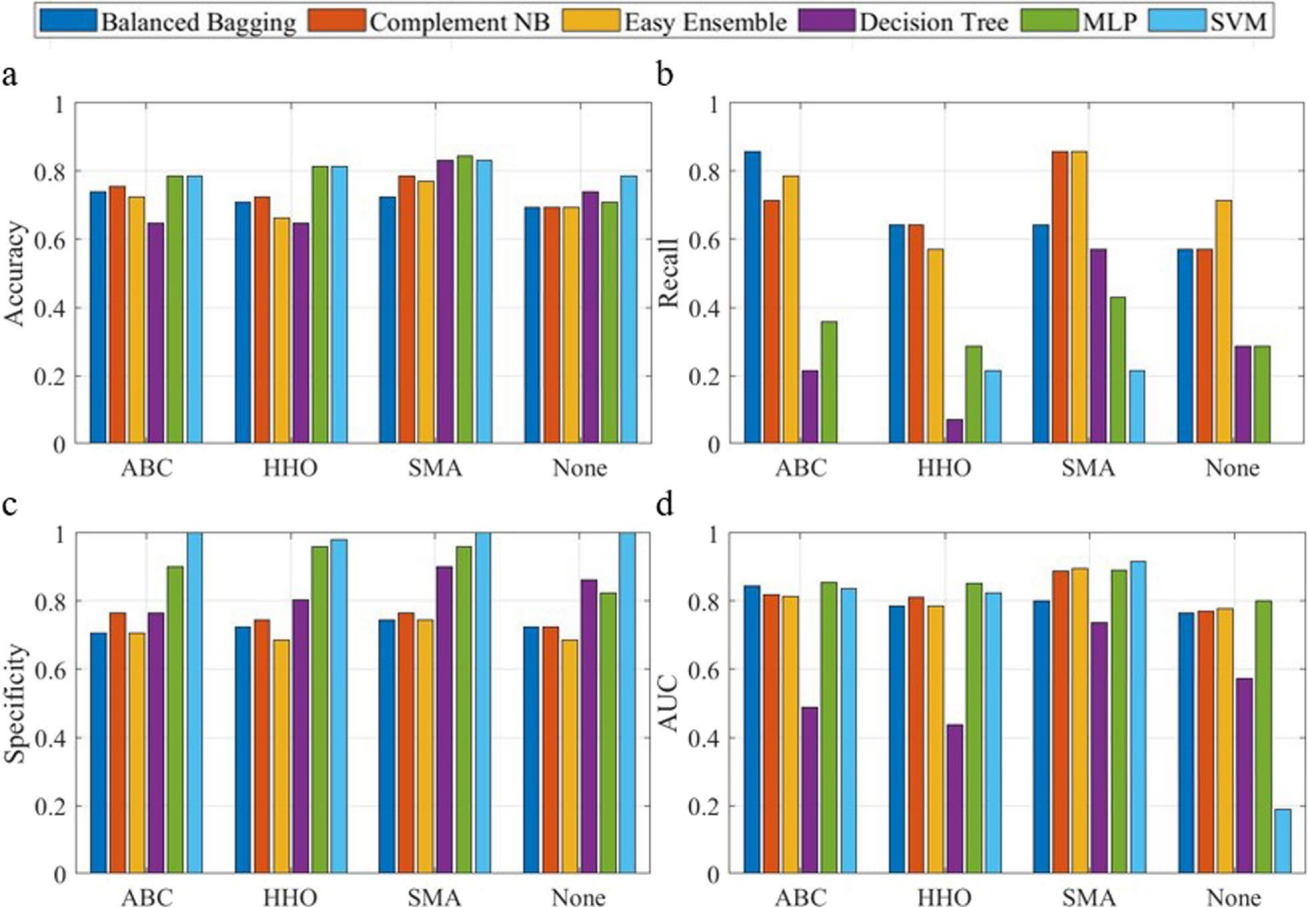
Model performance comparison using various classifiers for criteria II, illustrated for accuracy (**a**), recall (**b**), specificity (**c**) and area under the curve (**d**). (MLP: Multi-layer Perceptron; SVM: Support Vector Machine)

The model performance with various classifiers (Balanced Bagging, Complement NB, and Easy Ensemble) was compared with traditional models such as SVM, Decision Tree, and Multi-layer Perceptron (MLP) (Figs. 7 and 8) for the classification of fall risk based on Criteria I and II. The above traditional classifiers had a high specificity compared to Balanced Bagging, Complement NB, and Easy Ensemble. Meanwhile, they also exhibited quite low recall values, rending a smaller AUC. This trend was observed across both models for Criteria I and II, emphasizing the effectiveness of the implemented classifiers in handling imbalanced data for fall risk classification in this study.

### Explainability using SHAP

**Fig. 9:**
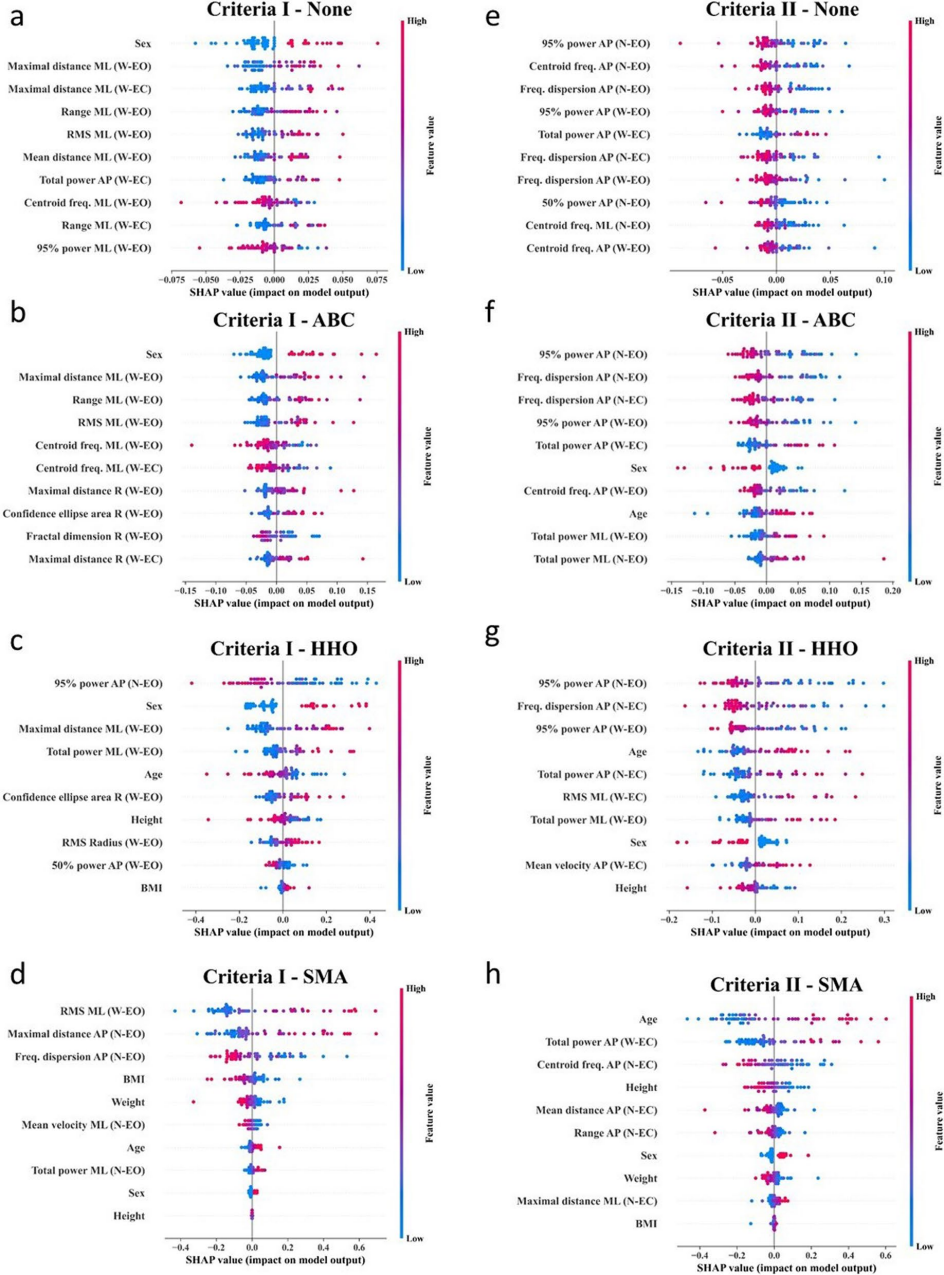
The SHAP summary visualization of the proposed model. The higher SHAP value of a feature corresponds to the higher prediction and feature importance for the different machine learning models were listed top-down

Figure 9 illustrates representation of the SHAP summary plots. The features are arranged in descending order of significance, while the SHAP values are displayed along the x-axis. The greater the distance from the vertical line at x = 0, the more significant the influence on the output prediction. Values situated to the left tend to steer the prediction toward an elevated risk of falling. The vertical lines, consisting of dots, are adorned with various colors. Each participant is represented by a dot, with pink denoting a high value and blue representing a low value. This figure effectively depicts the most critical features and their respective impact ranges.

Generally, using SMA showed high SHAP values in limited numbers of parameters, while no feature selection did not highlight the SHAP values among different parameters. Several posturographic parameters were selected often, such as total A-P and 95% power A-P in the W-EC condition, frequency dispersion A-P in the N-EO condition, and the RMS M-L in the W-EO condition.

For Criteria I, a number of posturography parameters was identified as important for fall classification, with most of them from W-EO condition and in the M-L direction. For personal metrics, age and sex were the mostly selected parameters across several models. Older age was associated with higher fall risk with Criteria II. In contrast, the effect of sex was not consistent. For criteria II, the SMA had the largest AUC and the highest accuracy. This model had higher weights from personal metrics (age, body height, body weight and sex) and several posturographic features. It was noteworthy that, unlike with criteria I, the posturographic features with high SHAP values were from the N-EC condition, the mostly challenging task, and also from posturographic parameters in the AP direction.

## Discussion

Fall risk classification is important to initiate a person-centered approach to fall prevention in community-dwelling older adults [52]. Using computerized posturographic parameters provides a highly quantitative and objective measure without ceiling or floor effect as a classifier or predictor of falls. This is advantages in community-dwelling older adults, who may still function well in community despite gradually declined balance function compared to the young adults. Application of ML algorithms showed promising roles to incorporate these complex, non-linear and highly correlated posturographic parameters. However, the superiority of these parameters is yet to be confirmed to increase the discriminative ability in fall classification or prediction. Our study design used four standing conditions to challenge the trunk controls and enhance discrimination. Moreover, the attribution of the lumbar tracker trajectory parameters obtained in each standing condition to the models was illustrated through the XAI approach. We also document that choices of feature selection techniques and classifiers help optimize the performance, regarding the high numbers of features from four standing conditions and imbalance data.

### Classification criteria

Our study results showed that the discriminating ability of the ML models was related to the classification criteria used to classify fall risk among community-dwelling older adults. We chose two criteria, which were either an important risk factor for falls or a commonly used screening tool for fall risks [8, 49]. As we hypothesized, the discriminating ability varied with different criteria. Using criteria II according to a TUG test scores had good performance, comparable with or even superior to some studies with different study designs and ML methods [14, 15, 19]. It is not surprising because of multifactorial nature of falls, while TUG scores were correlated with balance and could be reflected by static posturographic features. The results agree with previous studies using static parameters to classify at-risk or no-risk groups for falls in older adults [53, 54]. The AUC was between 0.6 and 0.9 with different posturographic parameters in a group of community-dwelling older adults, half of whom had a TUG larger than 13.5 s [54]. In contrast, the AUC was less than 0.7 in a study using future fall events to define fall risks. It is noteworthy that we used 10 s as a cutoff value, which was lower than the previously proposed values for high risk of falls. Our rational was based on a low proportion of our participants with scores higher than 12 s, and the average TUG score for the previous fallers was 10 s. The criterion was proposed in some studies among healthy community-dwelling older adults [4]. Presumably, using 10 s would be associated with a looser criterion to define fall risk. However, our models achieved a good performance, implying the effectiveness of the posturographic parameters in classifying these two groups.

In contrast, these posturographic parameters were much less effective in classifying fall risk according to a previous fall history. Since the nature of dependent variables for prediction or classification would influence the modeling performance [55], it is reasonable to say that these posturographic parameters obtained under different stance conditions can reflect the mobility balance better than a previous fall history through ML models [54]. It also echoes the fact that falls are the combined results of multiple intrinsic and extrinsic factors, not just balance ability [1]. Therefore, it is likely to increase the accuracy of ML when more comprehensive information related to risk factors for falls can be included.

### Comparison with traditional feature selection techniques and classifiers

The findings of our study indicated that the incorporation of feature selection techniques can significantly improve the accuracy and overall performance of classifiers across diverse classification tasks. We employed three feature selection models, ABC, SMA and HHO, based on meta-heuristic optimization. These models have demonstrated promising performance in previous research [42], and our results also confirmed their advantages over traditional methods, such as F-value and MI. The impact on the improvement of the model performance was mainly with criteria II. Using feature selection combined with complement NB can increase both TN and TP while reducing FP and FN, as demonstrated by the confusion matrix. Meanwhile, high FP was more than FN with Criteria I using feature selection reduced the TN while increasing or reducing TP. This could be attributed to a lower correlation between a fall history and posturographic parameters.

Additionally, to mitigate the challenges posed by imbalanced data, we employed three distinct classifiers, aiming to diminish bias towards the majority class, and thereby enhance the classification performance compared with traditional classifiers, such as Decision Tree, SVM and MLP. These three methods could achieve a high specificity, but a low recall. Notably, the utilization of the Complement NB classifier demonstrated promising potential as an effective approach for addressing classification problems.

### Explainablility

XAI represents a cutting-edge approach that seeks to establish transparency and trustworthiness in machine learning models [26]. This study contributes to the field by documenting the influence of posturographic parameters derived from various stance conditions and personal metrics on the model's performance. The main advantage of using SHAP is the transparency to identify which features are driving a model performance and how much each feature is contributing to the model. It has been used in health care [56], including one documenting the important factors attributing to mobility decline in the older adults [31]. The contribution of personal metrics was mostly in accordance with previous studies exploring risk factors of falls [2, 57]. This study used posturographic data from four stance conditions from the combination of stance width and eyes open/closed, and the results illustrated the significance of postural control strategies when individuals modify their stance width and rely on visual information cues. This investigation provides valuable insights into the role of these factors in shaping the model's decision-making process and enhances our understanding of the underlying mechanisms governing postural control in different contexts. Several posturographic and personal metrics demonstrated a high contribution to the fall risk classification, as determined by SHAP's output based on different feature selection approaches. It seemed that the posturographic parameters obtained during W-EO and W-EC conditions attributed more weights to the output with criteria I. In contrast, the parameters obtained during N-EC, the most challenging task, had higher attribution to the output with criteria II. It explained that a contribution of increased body sway in these challenging standing tasks, and a declined postural control could be classified effectively. The observation of higher SHAP values from posturographic parameters in the A-P direction with Criteria II was also in accordance with some previous studies using COP trajectories [54]. The results highlight the potential utility of these features in classifying fall risk and provide guidance for the development of more robust and accurate fall classification models among community-dwelling older adults.

### Limitation

Our study sample size was relatively small regarding a moderate effect size to classify falls [55]. The proportion of high-risk participants or actual falls was mostly lower than 30%, as observed in our study, and it resulted in an imbalanced dataset. Our sample size was larger than those in most of the previous studies [18, 19, 23], but a larger sample size should be aimed to ensure robust modeling for ML in the future. Second, this was a cross-sectional study, and a prospective and follow-up study design would be helpful to determine the predictive validity of these posturographic data. Third, the study mainly focused on the discriminative ability of the posturographic parameters. Since the mechanism underlying fall risk was complex, there were needs to collect more information to build models with higher predictive ability.

## Conclusion

Despite the importance of fall risk screening in the older adults, its implementation in the healthcare process is challenging [58]. The balance control problem is one of the major contributing factors to falls and may change with aging, medication, or acute illness. A system such as computerized posturography provides higher quantitative and objective information about trunk stability responding to different stance conditions with easily standardized procedures, autonomic recording, and digitalized data. The incorporation of an appropriate ML algorithm and XAI approach facilitates an autonomous evaluation with high accuracy and a transparent model for balance ability classification. However, it seems that using these parameters alone were not adequate for fall risk classification.

## Data Availability

The datasets used and/or analyzed during the current study are available from the corresponding author on reasonable request.

## Abbreviations

A-P: Anterior–posterior
ABC: Artificial Bee Colony
AI: Artificial intelligence
AUC: Area under the curve
BMI: Body mass index
COP: Center of pressure
FN: False negatives
FP: False positives
GM: Geometric Mean
HHO: Harris Hawk Optimization
MI: Mutual Information
ML: Machine learning
MLP: Multi-layer Perceptron
M-L: Medial–lateral
N-EC: Feet together with eyes closed
N-EO: Feet together with eyes open
NB: Naïve Bayes
ROC: Receiver’s operating characteristic
SHAP: SHapley Additive exPlanations
SMA: Slime Mould Algorithm
SVM: Support Vector Machine
TD_L_: Trunk displacements of the lumbar tracker
TN: True negatives
TP: True positives
TUG: Timed-up-and-go
W-EC: Feet apart with eyes closed
W-EO: Feet apart with eyes open
XAI: Explainable artificial intelligence (XAI)
